# “A cross-sectional study of burnout among Australian general practice registrars”

**DOI:** 10.1186/s12909-023-04043-4

**Published:** 2023-01-20

**Authors:** Rebekah Hoffman, Judy Mullan, Andrew Bonney

**Affiliations:** University of Wollongong, Wollongong, Australia

**Keywords:** Burnout, Parenting, Education, Medicine

## Abstract

**Objective(s):**

To identify if gender and parenting factors are associated with burnout in Australian general practice (GP) registrars.

**Design:**

Cross sectional study. The main outcome measure was the Maslach Burnout Inventory, included as part of the GPRA (General Practice Registrars Australia) biannual online survey.

Participants: GP registrars, 2019 cohort, undertaking fellowship training in Australia.

**Results:**

In 2019 a total of 366 GP registrars completed the online survey. Over 75% of registrars experienced moderate to high levels of burnout (emotional exhaustion scale). Several demographic factors were associated with an increased risk for reporting higher levels of burnout. Increasing age was associated with lower levels of personal accomplishment (*P*-value < 0.01), being female was associated with higher levels of emotional exhaustion (*p*-value < 0.001) and increasing numbers of children were associated with lower levels of burnout, independent of hours worked (*p*-value < 0.001).

**Conclusion:**

This study suggests that being a parent is associated with a reduced risk of burnout, irrespective of hours worked. However, being female and increased age were associated with increased levels of burnout. With increasing numbers of females entering medical training, and the decreasing desirability of general practice training, this paper reviews the complexities around parenting during training and associations with burnout. There is a need to examine this interaction further to understand the causation for these findings, and to ensure appropriate policies, opportunities and workplace supports are developed to ensure GP training is optimised to attract and support the next generation.

## Introduction

Australian doctors have higher rates of stress and burnout than the general population [1]. Stress and burnout appear especially prevalent in junior doctors [2], across both general practice and hospital-based training programs [3]. Burnout can lead to increased anxiety, increased risk of medical errors and increased risk of harmful behaviours such as excessive alcohol intake [4]. Specific demographic factors have been identified both at an individual level and at a systems level that place junior doctors at risk. Data demonstrates female doctors have an increased level of both psychological distress and depression compared to males [1, 2]. At a systems level, prolonged working hours and lack of flexibility in the workplace leads to increased levels of stress and burnout for both male and female junior doctors [3, 5]. The Maslach Burnout Inventory describes three burnout dimensions. Firstly depersonalisation, where there is an apathetic or removed attitude towards work and one’s patients or colleagues. Secondly, emotional exhaustion, where work demands lead to increased fatigue. Finally, personal accomplishment, where there are reduced feelings of success and effectiveness within the workplace [5, 6]. The sequence in which these dimensions occur differs in different populations [7]. It is considered that the early identification of behaviours, or triggers for burnout having the potential to lead to the development of strategies that reduce burnout and depression [5, 7].

The demographic profile of medical students, and in turn general practice registrars, has changed over the last decade. There are increasing numbers of females entering and training in medicine, with females having made up over 50% of the medical graduates since the 1990s, both in Australia [8] and internationally [9]. General practice has traditionally been viewed as a family friendly career, attracting larger cohorts of female doctors [10]. Specialty training often coincides with child rearing plans, and decisions prioritising career versus family have been described to lead to increased stress with both work and life implications [11–15]. The authors have not been able to identify any research that has explored the experiences of parenting and stress and burnout during General Practice training years in Australia. This study seeks to address some of these unexplored factors and to examine if there is an association between gender, having children and burnout in Australian general practice registrars.

## Methods

### Study design and setting

This cross-sectional study was conducted with general practice registrars in Australia in 2019. These doctors were invited via email to complete a questionnaire through General Practice Registrars Australia (GPRA), an independent organisation representing trainees working towards fellowship as a General Practitioner. The questionnaire was completed online, as part of GPRA’s ongoing studies. The GPRA study is conducted on a bi-annual schedule. Each iteration of the survey is reviewed by a group of registrars prior to release. The GPRA questionnaire contains a series of standard questions pertaining to contracts, income, and hours, and then a series of additional questions, nominated by registrars. The questions relating to this study were included as part of the 2019 additional questions and are described in more detail below.

Data were deidentified by GPRA staff prior to analysis. All General Practice registrars in Australia on any training pathway were eligible to participate.

Ethics approval was obtained through the Human Research Ethics Committee at the University of Wollongong, 2019/368.

### Measures

Measures from the 83-item 2019 GPRA questionnaire, completed In 2019, the abbreviated Maslach Burnout Inventory (aMBI) [16] was included in the GPRA survey. The aMBI is a nine-item scale that assesses burnout across three subscales: emotional exhaustion, depersonalisation, and personal accomplishment. The emotional exhaustion subscale identifies burnout due to emotional depletion. The depersonalisation subscale describes an impersonal, or a detached response to the patient – doctor treatment relationship. The personal accomplishment subscale describes the individual’s impression of their competence. Each subscale is assessed by three questions, and each question is scored over a 7-point Likert-type response item. For emotional exhaustion and depersonalisation, a higher score demonstrates increased levels of burnout. For personal accomplishment, the score is inverted, and a higher score is assessed as indicating a lower level of burnout. Validation studies have considered moderate burnout to occur at depersonalisation scores above 4, emotional exhaustion scores above 7 and personal accomplishment scores below 14. High burnout is considered at depersonalisation scores above 7, emotional exhaustion scores above 11 and personal accomplishment scores below 12 [17]. No to low burnout levels are validated to occur at the remaining scores [17]. The validity and reliability of the aMBI has been established internationally [17]. The study variables of gender and number of dependents were derived from the 83-item 2019 GPRA questionnaire, along with age and work arrangements as potential confounders.

### Data analysis

The internal consistency for each aMBI scale was calculated using Cronbach’s alpha. Reliability scores of > 0.7 were considered to be acceptable; [18, 19] higher scores demonstrating greater internal consistency. Initial bivariate analyses were undertaken to test the relationships between the aMBI subscales and gender, number of dependents, age, and work arrangements respectively. To aid interpretation, aMBI sub-scale scores were categorised according to accepted severity levels, and age and work arrangements were categorised to age brackets and no work, part- or- full time employment. Associations between aMBI sub-scale burnout score categories and the independent variables were assessed using the chi-square test. Multi-variate regression models were then fit to assess independent associations between burnout scores and gender, number of dependents, age and hours worked. The raw aMBI sub-scales scores were as the dependent variables (rather than categorised) in linear regression models to facilitate model convergence and interpretation. The age variable was grand mean centred, again to facilitate an interpretable intercept for all models, i.e. the intercept at the mean age of the participants, not zero. All independent variables were included in all models simultaneously due to their theoretical associations with burnout. A *p*-value of < 0.05 was considered statistically significant. Data analysis was performed using the ezR package [20] for the R-computing environment [21].

## Results

### Demographics

Completed questionnaires were returned by 366 general practice registrars. There were 4008 trainees on the AGPT (Australian General Practice Training) pathway in 2019, this survey represents 9.7% of registrars training at the time of the survey. The majority of respondents to the survey were female (65%, *n* = 239) and worked full time (66.7%, *n* = 244) (Table 1). The largest cohort of registrars were 30-34 years of age, ranging from under 30 years (28%, *n* = 107) to over 60 years (*n* = 1). Most registrars had no children (*n* = 202, 55%), while 18% (*n* = 66) had one child, 19% had two children (*n* = 69) and 8% had more than three children (*n* = 29). The demographic data for the study cohort, is fairly consistent with the demographic data from the AGPT population of the same year, which suggested that 60% of the trainees in 2019 were female, 72% worked full time, 20% were over forty years of age, and four percent were over fifty years of age [22].

**Table 1:** Demographics

Characteristics	N	Frequency (%)
GENDER		
Male	124	33.87
Female	239	65.31
Prefer not to say	3	0.82
AGE		
under 30 years old	103	28.06
30–34 years old	119	32.71
35–39 years old	70	19.07
40–44 years old	37	10.08
45–49 years old	20	5.45
50–54 years old	12	3.27
55–59 years old	4	1.09
60 + years old	1	0.27
DEPENDANTS		
No children	202	55.19
1 child	66	18.04
2 children	69	18.85
3 + children	29	7.92
WORK ARRANGEMENTS		
Fulltime	244	66.67
Part time	80	21.86
Not working	42	11.47

### Abbreviated Maslach Burnout Inventory bivariate analyses

Cronbach’s alpha for aMBI subscales was reported as Depersonalisation 0.69, Emotional Exhaustion 0.82, and Personal Accomplishment 0.57. High burnout levels were reported across all subscales of the aMBI at rates of 48.2% for emotional exhaustion, 27.0% for depersonalisation and 24.0% for personal accomplishment. Moderate burnout levels were reported at rates of 25.6% for emotional exhaustion, 21.8% for depersonalisation and 19.6% for personal accomplishment. There was a statistically significant association with gender and emotional exhaustion (*p* = 0.03). There were no statistically significant differences in gender for the depersonalisation and personal accomplishment subscales. There were significant associations between age and emotional exhaustion (*p* = 0.02), and number of dependants and both emotional exhaustion (*p* =  < 0.001) and depersonalisation (*p* =  < 0.001). Similar findings were not seen for working hours, where there was no significant association across any scale of the aMBI. Burnout results are demonstrated in Table 2, and the subscale analysis in Table 3.

**Table 2:** Abbreviated Maslach Burnout Inventory bivariate analyses

**Burnout totals**	aMBI subscale	Emotional Exhaustion	Depersonalisation	Personal accomplishment
	no to low burnout (n, %)	96 (26.2)	188 (51.2)	207 (56.4)
	moderate burnout (n, %)	94 (25.6)	80 (21.8)	72(19.6)
	high burnout (n, %)	177 (48.2)	99 (27.0)	88 (24.0)

**Table 3:** Abbreviated Maslach Burnout Inventory bivariate analyses, Subscale analysis

			Emotional Exhaustion	Depersonalisation	Personal accomplishment
**Gender**	no to low burnout, n (%)	Male	40 (10.9)	56 (16.3)	75 (20.3)
		Female	54 (14.6)	129 (34.4)	130 (35.4)
	moderate burnout, n (%)	Male	35 (9.6)	30 (8.1)	21 (5.7)
		Female	58 (15.7)	49 (13.3)	50 (13.6)
	high burnout n, (%)	Male	49 (13.2)	38 (10.2)	27 (7.3)
		Female	128 (34.8)	60 (16.5)	59 (16)
	*P*-value		0.03	0.26	0.48
**Age**			Emotional Exhaustion	Depersonalisation	Personal accomplishment
	no to low burnout, n (%)	under 30	16 (4.4)	45 (12.3)	59 (16.1)
		30–39	54 (14.8)	95 (25.9)	103 (28.2)
		40 +	25 (6.4)	46 (12.7)	43 (11.6)
	moderate burnout, n (%)	under 30	25 (6.9)	27 (7.4)	28 (7.7)
		30–39	51 (14)	41 (11.2)	33 (9)
		40 +	18 (4.6)	12 (3.2)	11 (3)
	high burnout (%), n	under 30	62 (16.9)	31 (7.9)	16 (4.4)
		30–39	83 (22.6)	52 (14.2)	52 (14.4)
		40 +	31 (8.6)	17 (4.5)	19 (5.3)
	*P*-value		0.02	0.23	0.07
**Number of dependants**		Dependants	Emotional Exhaustion	Depersonalisation	Personal accomplishment
	no to low burnout, n (%)	No	32 (8.7)	85 (23.2)	105 (26.6)
		1	27 (7.5)	36 (9.9)	40 (11.1)
		2 +	35 (9.4)	68 (18.5)	60 (16.4)
	moderate burnout, n (%)	No	50 (13.7)	49 (13.4)	44(12)
		1	16 (4.4)	13 (3.3)	12 (3.2)
		2 +	28 (7.6)	16 (5)	15 (4.4)
	high burnout, n (%)	No	120 (32.8)	68 (18.5)	53 (14.5)
		1	22 (6)	16 (5)	13 (3.5)
		2 +	35 (10.1)	15 (4.2)	20(5.5)
	*P*-value		** < 0.001**	< 0.001	0.38
**Work arrangements**		Hours worked	Emotional Exhaustion	Depersonalisation	Personal accomplishment
	no to low burnout, n (%)	Full time	61 (24.8)	118 (48.4)	141 (57.7)
		Part time	26 (32.5)	47 (58.7)	43 (53.7)
		Not working	8 (21.9)	22 (53.7)	22 (53.6)
	moderate burnout, n (%)	Full time	68 (27.6)	54 (22)	43 (17.5)
		Part time	15 (18.7)	17 (21.2)	17 (21.2)
		Not working	11 (26.8)	9 (22)	12 (29.3)
	high burnout, n (%)	Full time	117 (47.7)	73 (29.6)	61 (24.8)
		Part time	39 (48.7)	16 (19.8)	20 (24.8)
		Not working	21 (51.2)	10 (24.3)	7 (17)
	*P*-value		0.4	0.45	0.44

### Multivariate linear regression analysis

Gender and number of dependants were independently associated with emotional exhaustion scores in multivariate linear regression models. Male gender was associated with lower scores in the emotional exhaustion subscale (*p* =  < 0.001). The number of dependants had an inverse association, where the more dependants that the doctor had, the lower the reported scores for emotional exhaustion (*p* =  < 0.01). There were no independent associations between emotional exhaustion and age or number of hours worked.

The number of dependants was independently associated with depersonalisation scores in an inverse relationship, where the more children the doctor had, the lower the scores for depersonalisation (*p* =  < 0.001). There was no statistically significant relationship seen for any of the other variables.

Age was inversely independently associated with personal accomplishment (*p* =  < 0.01). The number of dependants was independently associated with personal accomplishment, where the more children the doctor had, the higher the scores for personal accomplishment (and lower levels of burnout) (*p* = 0.02). There were no significant associations with the burnout scores and hours worked. The linear regression models are summarised in Table 4.

**Table 4:** Abbreviated Maslach Burnout Inventory linear regression models (fully adjusted)

Independent variables	Co-efficient	Standard error	T-value	*P* values
**Emotional Exhaustion** R^2^ 0.08				
Intercept	11.22	0.61	18.23	< 0.001*
Gender^b^	-1.63	0.47	-3.50	< 0.001*
Age^a^	-0.11	0.19	-0.59	0.55
Dependants	-0.86	0.26	-3.29	< 0.001*
Hours worked	0.05	0.34	0.14	0.88
**Depersonalisation** R^2^ 0.06				
Intercept	4.79	0.55	8.65	< 0.001*
Gender^b^	0.35	0.42	0.89 4	0.40
Age^a^	-0.11	0.17	-0.64	0.51
Dependants	-0.85	0.24	-3.62	< 0.001*
Hours worked	0.18	0.30	0.59	0.56
**Personal Accomplishment** R^2^ 0.03				
Intercept	14.56	0.41	35.49	< 0.001*
Gender^b^	0.53	0.31	1.71	0.09
Age^a^	-0.35	0.12	-2.75	< 0.01*
Dependants	0.40	0.17	2.26	0.02*
Hours worked	-0.08	0.23	-0.37	0.71

^a^Mean age was 31.4 years

^b^Reference Female

^*^Statistically significant results

## Discussion

This study aimed to investigate the relationship between the previously unexplored factors of gender and having children and burnout in Australian general practice registrars, controlling for confounding factors such as age and hours worked. To the best of our knowledge, this has not been previously explored in the literature. There were several key findings. Burnout rates were high across all subscales of the aMBI. Emotional exhaustion was demonstrated to occur at higher levels compared to depersonalisation and personal accomplishment. Associations were noted between burnout and a number of demographic factors. For instance, increasing age was associated with higher levels of burnout within the subscale of personal accomplishment. Increased number of dependants was associated with lower levels of burnout, independent of other factors including number of hours worked.

We observed across all ages and genders that emotional exhaustion was the most common subscale which demonstrated moderate to high levels of burnout. This is consistent with the literature which demonstrates that in workplaces where there are high levels of stress such as general practice, emotional exhaustion can be seen as a precursor to depersonalisation and worsening burnout [23]. The high demands on cognitive load leads to mental and physical exhaustion, which in turn can lead to depersonalisation as a coping strategy for the exhaustion [23]. This is reflected in this study, where emotional exhaustion levels are higher, potentially as a precursor to depersonalisation and worsening personal accomplishment. This gives an opportunity for GP colleges, regional training organisations, and local training practices to work to identify strategies to reduce emotional exhaustion [23]. This could be an opportunity to improve the desirability of general practice training, and both increase the numbers of applicants into GP training, and reduce early career burnout, through the development of emotional regulation training and strategies such as increasing teamwork and reducing external pressures [24]. This may lead to reduced burnout and increased registrar retention [25].

Increasing age was associated with lower personal accomplishment scores, which were independent of other factors investigated as part of this study. A study by Cook et al. (2013) discussed personal accomplishment and burnout in General Practice Registrars. They identified that anxiety as a result of clinical uncertainty in patient encounters, and a reluctance to disclose this uncertainty were associated with being at higher risk of burnout [26]. This explanation in the literature may be applicable to our cohort. It may be that GP registrars that worked for an increased number of years, and may be more senior in age, experience a cumulative effect of emotional and mental load facing clinical uncertainty and the breadth of knowledge required in general practice. This provides opportunity for further research to identify the cause of this finding and to develop policy and training strategies to better support mature trainees.

Males and females both experienced high levels of burnout in the current study. Within each subscale of emotional exhaustion, depersonalisation, and personal accomplishment there were some differences. For instance, females were more likely to experience emotional exhaustion, whereas within the depersonalisation and personal accomplishment subscales there were similar levels of burnout reported across genders. Previous research has attributed increased levels of burnout in females to demands on work life balance and an increased emphasis on parenting and home life [27, 28]. The increased emotional exhaustion levels reported by females in our study may be of concern, given that emotional exhaustion has been identified as a precursor to worsening levels of burnout [23]. With more females entering general practice training [22], and higher numbers of females in this study reporting emotional exhaustion, the reasons for this and protective supports available to reduce burnout should be examined.

Having children may impact on career, by way of delaying or changing career goals [29, 30], reducing potential income [31], and placing increased pressure on work life balance [32, 33]. However, this study demonstrated that having children was associated with lower levels of burnout, and this was increasingly protective with increased numbers of children. This has also been demonstrated in other careers, where it has been argued that having a career and a family are mutually beneficial in preventing burnout, with a family buffering the impact that workload has on burnout [34]. This association may have a number of causes, including; that parenting is associated with increased learned resilience, or that registrars who are experiencing lower levels of burnout in training are more likely to plan to increase the size of their family. There are other individual registrar factors which may lead to lower levels of burnout and increased family size, such as home based and external supports, and family size preferences [35]. This finding points to the diverse intricacies around child rearing during career planning and professional training. It provides an opportunity to do further research into this complexity and examine the interactions between parenting, training demands, workplace supports and provide overarching policies and individual supports to trainees.

### Limitation of study

The limitations of this study include the inability to determine the return rate; as an annual survey, formal records of the number of invitations sent to GP registrars were not kept limiting the ability to calculate a return rate. This has limitations in our ability to generalise these results across the wider GP Registrar cohort. The cross-sectional survey design is a limitation in identifying causation. The paper only considers a small number of potential independent variables and future research should look at alternate study designs, including prospective cohort designs, to further explore the findings found in this study.

## Conclusion

There is a current declining desirability of general practice as a training prospect and increasing feminisation of the workforce. This study adds to the literature, that being a parent is associated with a reduced risk of burnout, irrespective of hours worked. However, we found that being female and increased age were associated with increased levels of burnout in some domains. This points to a greater complexity than previously described regarding burnout and the parenting and medical training interaction. This interaction should be examined further to understand the causation for these findings, and to ensure appropriate policies opportunities and workplace supports are developed to ensure GP training if future proofed to the next generation.

## Data Availability

The data that support the findings of this study are available from General Practice Registrars Australia but restrictions apply to the availability of these data, which were used under license for the current study, and so are not publicly available. Data are however available from the authors upon reasonable request and with permission of General Practice Registrars Australia. The point of contact for Data Availability Request is the corresponding author; Dr Rebekah Hoffman (rhoffman@uow.edu.au).
